# A Predictive Coding Framework for Understanding Major Depression

**DOI:** 10.3389/fnhum.2022.787495

**Published:** 2022-03-03

**Authors:** Jessica R. Gilbert, Christina Wusinich, Carlos A. Zarate

**Affiliations:** Experimental Therapeutics and Pathophysiology Branch, National Institute of Mental Health, National Institutes of Health, Bethesda, MD, United States

**Keywords:** major depression, predictive coding, mismatch negativity, reward processing, prediction errors, ventral striatum

## Abstract

Predictive coding models of brain processing propose that top-down cortical signals promote efficient neural signaling by carrying predictions about incoming sensory information. These “priors” serve to constrain bottom-up signal propagation where prediction errors are carried via feedforward mechanisms. Depression, traditionally viewed as a disorder characterized by negative cognitive biases, is associated with disrupted reward prediction error encoding and signaling. Accumulating evidence also suggests that depression is characterized by impaired local and long-range prediction signaling across multiple sensory domains. This review highlights the electrophysiological and neuroimaging evidence for disrupted predictive processing in depression. The discussion is framed around the manner in which disrupted generative predictions about the sensorium could lead to depressive symptomatology, including anhedonia and negative bias. In particular, the review focuses on studies of sensory deviance detection and reward processing, highlighting research evidence for both disrupted generative predictions and prediction error signaling in depression. The role of the monoaminergic and glutamatergic systems in predictive coding processes is also discussed. This review provides a novel framework for understanding depression using predictive coding principles and establishes a foundational roadmap for potential future research.

## Introduction

The predictive coding framework suggests that the brain functions to minimize surprise and uncertainty by actively generating explanations for encountered stimuli (Friston, 2009). The framework is rooted in Bayesian probability theory and the so-called *Bayesian brain hypothesis* (Knill and Pouget, 2004) that conceptualizes perception as a constructive process that uses internal or generative models to encode prior beliefs about sensory inputs and their causes. Generative models help an individual formulate predictions about incoming sensory information that are tested against incoming sensory inputs and produce prediction errors. Prediction errors, in turn, are used by the brain to revise its model of the world by updating predictions in order to minimize prediction errors (Friston, 2010). Recent work has extended these ideas to cognitive phenomena related to interoception (Seth, 2013), including the shaping of emotions (Seth and Friston, 2016; Clark et al., 2018) and the development of depression (Barrett et al., 2016; Kube et al., 2020).

Interoception, broadly defined as the sense of the physiological condition of the body (Craig, 2002), is proposed to be the sensory consequence of allostasis, the regulation of metabolism and bodily states (Barrett et al., 2016). Several recent reviews have focused on the role of predictive processes related to interoception in the etiology and pathophysiology of depression (Barrett and Simmons, 2015; Barrett et al., 2016; Stephan et al., 2016; Eggart et al., 2019). Rather than focusing on interoceptive processes, this review examines the electrophysiological and neuroimaging evidence regarding predictive coding deficits in exteroception in depression, focusing on sensory deviance detection and reward processing deficits that accompany major depressive disorder (MDD).

Bayesian models can be used to inform our understanding of neural and circuit-level dysfunction concomitant with psychiatric conditions such as MDD because they relate formal information-processing algorithms to underlying neural signals (O’Reilly et al., 2012). In current models of Bayesian brain updating, for example, predictions are thought to be carried by descending feedback from deep pyramidal cortical layers and to interact with ascending, feedforward prediction error signals from superficial cortical layers (Rao and Ballard, 1999; Friston and Kiebel, 2009; Bastos et al., 2012; Shipp et al., 2013). These prediction error signals serve to update an individual’s expectations, with the precision or confidence placed in prediction errors associated with the synaptic gain or efficacy of superficial pyramidal cell signaling. From a theoretical standpoint, a model such as this can help elucidate cardinal differences between individuals with MDD and healthy participants because the fitting of experimental data to such a model can provide a mechanistic understanding of differences between groups. For example, such models could help an investigator test whether deficits in MDD are related to faulty internal generative models and resultant prediction error signaling associated with incoming sensory information. These models could also be used to make inferences about where prediction error signals originate in the cortex, and these regions can be probed to determine whether activity in a given region is associated with specific features of depressive symptomatology.

This review will highlight the electrophysiological and neuroimaging evidence for disrupted predictive processing in MDD, conceptually framing the discussion around Bayesian models of uncertainty and how disrupted generative predictions about the sensorium might lead to depressive symptomatology, including anhedonia and negative bias. This review will highlight studies of sensory deviance detection and reward processing in particular, describing research evidence for both disrupted predictions and prediction error signaling in MDD. Gaps in the literature where further research is warranted will also be discussed. Finally, the role of the monoaminergic and glutamatergic systems in generating these signals will be examined. This review provides a novel framework for understanding MDD using predictive coding principles and establishes a foundational roadmap for potential future research.

## Disrupted Sensory Deviance Detection in Major Depressive Disorder

Sensory deviance detection—broadly defined as the ability to detect deviant stimuli while attending to a stream of incoming sensory information—is thought to reflect pre-attentive sensory processing (Schröger, 1998; Restuccia et al., 2006; Czigler, 2007). One technique for studying pre-attentive change detection involves using an oddball paradigm where a series of frequent stimuli (e.g., tones of a specific pitch “standard”) are occasionally interrupted by less-frequent stimuli (e.g., tones of a higher pitch “deviant”). These kinds of paradigms have traditionally been collected using electrophysiological techniques such as electroencephalography (EEG) and magnetoencephalography (MEG) because the temporal components of event-related potentials (ERPs) generated in response to these stimuli have consistent response characteristics and well-documented neural generators (Garrido et al., 2009). In particular, a negative component in the event-related waveform is elicited by deviant relative to standard stimuli, which has been termed the mismatch negativity (MMN) response. The MMN response is considered an index of change detection processes (Näätänen et al., 2012) and, within a prediction coding framework, is thought to represent prediction error signaling (Friston, 2005). Generators of MMN electrophysiological signatures have been localized to primary and secondary auditory, visual, somatosensory, and olfactory cortices, and they have also been localized to higher-order regions, including the frontal cortex (Garrido et al., 2009).

Studies of the MMN response in MDD patients have reported mixed findings regarding waveform topographical changes accompanying MDD (see Table 1). The amplitude and latency of the characteristic MMN response—which occurs at approximately 100–250 ms after stimulus onset—has been measured in individuals with MDD relative to healthy participants. While some studies have reported that the MMN amplitude is attenuated in currently medicated and unmedicated MDD patients relative to healthy participants (Takei et al., 2009; Qiu et al., 2011; Qiao et al., 2013; Chen et al., 2015; Tseng et al., 2021), other studies have reported that the MMN amplitude is increased in unmedicated MDD patients (Kähkönen et al., 2007; He et al., 2010). Other studies found hemispheric asymmetries in the MMN response in MDD patients, with reduced MMN amplitudes in the right but not the left hemisphere in medicated MDD patients compared to healthy participants (Hirakawa et al., 2017). Furthermore, other studies reported MMN latency differences in MDD, with patients demonstrating slower peak MMN latencies than healthy participants (Qiao et al., 2013; Tseng et al., 2021). Finally, a small number of studies reported MMN amplitude changes for specific sensory features of oddball stimuli (e.g., timbre and tone duration), but not others (e.g., pitch, intensity, or location) in MDD patients (Mu et al., 2016; Tseng et al., 2021). Taken together, these findings provide preliminary evidence that MDD is accompanied by an inability to accurately predict forthcoming sensory information, though significant inconsistencies exist with regard to whether the MMN amplitude is larger or smaller and whether it is shifted in time compared to healthy participants.

**TABLE 1:** Sensory deviance detection disruptions in MDD.

Authors	Major findings—MMN	Sample size and characteristics	Medication status	Methodology
Chen et al. (2015)	↓ MMN amplitude in first-episode and recurrent MDDs; no association between depression severity and MMN amplitudes; P3a amplitude negatively associated with depression severity in both MDD groups	45 first-episode MDD, 40 recurrent MDD, 46 HC	Medicated	EEG
Takei et al. (2009)	↓ MMN amplitude in MDDs; no association between depression severity and MMN amplitude/latency	14 MDD, 19 HC	Medicated	MEG
Hirakawa et al. (2017)	↓ MMN amplitude in MDDs in right but not left hemisphere, reduced MMN latencies in both hemispheres; no association between depression severity and MMN amplitude/latency	20 MDD, 36 HC	Medicated	MEG
Tseng et al. (2021)	↓ MMN amplitude and prolonged latency in first-episode/early stage MDDs for duration but not frequency deviants, ↓ MMN amplitude only for duration deviants in recurrent MDDs; no association between depression severity and MMN amplitude/latency	Meta-analysis of studies including 339 MDD, 343 HC	Mixed status	EEG and MEG
Qiao et al. (2013)	↓ MMN amplitude and prolonged latency in MDDs for increment but not decrement deviants; no association between depression severity and MMN amplitudes	20 first-episode MDD, 20 HC	Unmedicated	EEG
Qiu et al. (2011)	↓ MMN amplitude in MDDs for long-duration but not short-duration deviants; no association between depression severity and MMN amplitudes	24 first-episode MDD, 24 HC	Unmedicated	EEG
He et al. (2010)	↑ MMN amplitude in MDDs only compared to other groups; no association between depression severity and MMN amplitudes or latencies	22 MDD, 19 BPD, 22 comorbid MDD/BPD, 32 HC	Unmedicated	EEG
Kähkönen et al. (2007)	↑ MMN amplitude in MDDs for 10% but not 20% frequency change deviants in EEG but not MEG; P1 latency decrease negatively associated with depression severity	13 MDD, 12 HC	Unmedicated	EEG and MEG
Mu et al. (2016)	↑ MMN amplitude in MDDs for timbre but not pitch, location, intensity, slide, or rhythm deviants; no association between depression severity and MMN amplitudes or latencies	20 MDD, 20 HC	Unmedicated	EEG
Kim et al. (2020)	No differences in MMN amplitude between MDD and HC; ↓ MMN amplitude in BD compared to HC	27 MDD, 29 BD, 33 HC	Medicated	EEG

*BD, bipolar depression; BPD, borderline personality disorder; EEG, electroencephalography; HC, healthy control; MEG, magnetoencephalography; MDD, major depression; MMN, mismatch negativity.*

In addition to examining differences in MMN amplitudes and latencies, an important clinical question is whether differences in pre-attentive change detection are associated with depressive symptomatology in MDD. Several studies have examined whether changes in components elicited during an oddball task are associated with severity of depressive symptoms or other clinical measures of functional outcomes. For example, a recent study comparing both medicated and unmedicated MDD patients to healthy participants found that clinical measures of functional outcomes for MDD patients were associated with MMN source activity in regions including the anterior cingulate and the inferior and middle frontal gyri, though no significant differences in MMN amplitudes were noted in MDD patients compared to healthy participants (Kim et al., 2020). Other studies that did not source-localize MMN generators found no significant associations between severity of depressive symptoms and MMN amplitudes or latencies (He et al., 2010; Mu et al., 2016; Tseng et al., 2021), though earlier and later waveform components, such as the attenuation of the P1 (Kähkönen et al., 2007) and the amplitude of the P3a (Chen et al., 2015), have been associated with clinical characteristics such as severity of depressive symptoms and the number of depressive episodes reported by patients. The P1 is a positive ERP waveform component occurring approximately 100 ms after stimulus presentation and thought to reflect initial sensory attentional processing, while the P3a is a positive component occurring approximately 250–280 ms after stimulus presentation that localizes to fronto-central electrode sites and reflects attentional orienting and novelty detection processes. Taken together, the evidence suggests that gross changes in MMN response characteristics such as amplitude and latency are indeed associated with clinical measures reflecting the severity of depressive symptoms. Further research should continue to explore the relationship between source-localized generators of the MMN signal and depressive symptomatology, given that source-localized MMN response estimates in regions such as the anterior cingulate and inferior frontal gyrus could provide a stronger index of the severity of depressive symptoms compared to waveform characteristics alone.

Inconsistencies in MMN response findings in MDD may be due to several factors, including differences in the sensory modality under study, manipulations regarding what constitutes standard and deviant stimuli, sample sizes, and recruitment criteria for MDD samples. For example, some studies recruited drug-free patients only, while others included a mixture of medicated and unmedicated patients. Special caution should be exercised in interpreting studies where the samples include medicated patients, particularly those in which patients are taking selective serotonin reuptake inhibitors (SSRIs), the most widely prescribed antidepressants. This is because serotonin (5-hydroxytryptamine, 5-HT) is thought to play an important role in salience detection (see “Antidepressant Drugs and Predictive Processes,” below). The heterogeneity of findings regarding deficits in the MMN response in MDD across studies may also be explained by the underlying heterogeneity of MDD symptomatology. For example, the MMN response has been hypothesized to index cognitive decline across different psychiatric disorders (Näätänen et al., 2012), suggesting that dysregulated sensory change detection, as indexed by the MMN response, might have prognostic importance in MDD.

As research expands our understanding of the sensory deviance detection deficits that accompany MDD, it is important to keep in mind the potential applications of this work. For example, a better understanding of the brain circuitry supporting prediction errors in sensory processing and their connectivity would improve our understanding of how feedforward and feedback signaling interact as well as illuminate the ways that these might be dysregulated in MDD. In addition, understanding the relationship between MMN signaling deficits and depression symptomatology could lead to the development of a simple, robust biomarker of symptom severity. Such work also fits within the larger Research Domain Criteria (RDoC) framework examing the relationship between neural circuitry disruption and dimensional symptomatology associated with mental disorders.

## Disrupted Reward Prediction and Prediction Error Signaling in Major Depressive Disorder

Reinforcement learning, the process by which behavior is modified through experiences with reward and punishment, offers a theoretical framework for studying the neural circuity supporting decision making under conditions of uncertainty (Schultz, 2006). Several lines of evidence now suggest that dysfunctional reinforcement learning processes and dysregulated reward circuity might underlie some symptoms of MDD (Pizzagalli, 2014). Anhedonia, or hyposensitivity to rewards, is a cardinal symptom of MDD and is associated with worse outcomes, including poor treatment response and greater prevalence of suicidal thoughts and behaviors (Eshel and Roiser, 2010; Spijker et al., 2010; Pizzagalli, 2014; Vrieze et al., 2014; Winer et al., 2016; Yaseen et al., 2016; Loas et al., 2018). Negative bias—a hypersensitivity to punishment and a bias in expectation of negative events—is another common feature of MDD (Gotlib, 1983; Eshel and Roiser, 2010; Rouhani and Niv, 2019). It is worth noting that, though conceptualized distinctly, neural correlates of anhedonia and negative bias may overlap and mutually influence depressive symptoms and differences in reward processing.

Reinforcement learning paradigms using monetary incentives provide an avenue for modeling brain circuitry disruptions in reward processing associated with anhedonia and negative bias. During such tasks, discrepancies between an expected reward and a given reward produce reward prediction errors (RPEs). The neural correlates underlying belief updating, during which a participant alters their framework to make more accurate predictions to subsequent trials, can be examined. Growing evidence suggests that key neural regions mediating RPE signaling include the lateral habenula, the ventral tegmental area (VTA), and the substantia nigra (Matsumoto and Hikosaka, 2007). The lateral habenula occupy a set of nuclei within the posterior-dorsal-medial region of the thalamus that are thought to have an important role in reward learning behavior [for a recent review of the circuity and functions of the lateral habenula, see Hu et al. (2020)]. The lateral habenula acts as a relay station by connecting the limbic forebrain with monoaminergic centers implicated in the pathophysiology of depression and has been proposed to participate in processing negatively valenced information (Yang et al., 2018b). Animal studies have demonstrated that lateral habenula neurons transmit RPEs in an inverted fashion (Matsumoto and Hikosaka, 2007, 2009a) and can suppress both activity in dopamine neurons (Christoph et al., 1986; Ji and Shepard, 2007) and motivated behaviors (Shumake et al., 2010; Friedman et al., 2011). While many lateral habenula neurons transmit information related to motivational salience (Matsumoto and Hikosaka, 2009b; Bromberg-Martin and Hikosaka, 2011), a subset of these neurons transmit information related to motivational value and exert control over selective positive RPE (i.e., signaling more reward than anticipated) and negative RPE (i.e., signaling less reward than anticipated) dopamine neurons (Matsumoto and Hikosaka, 2009b). Reward-related dopamine signals from the midbrain are broadcast to various regions of the cortex, including the striatum (particularly the nucleus accumbens), the prefrontal cortex, and the amygdala (Schultz, 2007; Niv and Montague, 2009).

Reward processing involves several distinct stages, and many studies have focused on the neural circuitry supporting the reward anticipation and feedback periods. Reward-related learning in particular is thought to occur through RPEs encoded by striatal dopamine signals (Schultz, 2016b). Several lines of evidence suggest that, compared to healthy participants, both medicated and unmedicated MDD patients have blunted RPE signaling within the ventral striatum during reward feedback (see Table 2; Zhang et al., 2013; Keren et al., 2018; Kumar et al., 2018), and EEG studies have consistently reported significant reductions in the feedback-related negativity (FRN) ERP component in medicated and unmedicated MDD patients compared to healthy participants (Keren et al., 2018). In addition, some studies have suggested that VTA-striatal connectivity is blunted in response to reward feedback in unmedicated MDD patients compared to healthy participants (Kumar et al., 2018). However, other researchers have found seemingly contradictory results regarding RPE signaling in the striatum. For example, Rutledge and colleagues (2017) found that ventral striatum RPE signaling did not significantly differ between medicated MDD patients and healthy participants, while a recent review identified discrepancies in blunting or lack of blunting of ventral striatum signals during RPEs in MDD patients (Yaple et al., 2021). Taken together, a growing consensus suggests that MDD is accompanied by changes in dopaminergic signaling that affect reward-related outcomes in the striatum, though significant inconsistencies remain regarding whether the striatal activation is blunted or increased in MDD.

**TABLE 2:** Reward prediction and prediction error disruptions in MDD.

Authors	Major findings—Reward signaling	Sample size and characteristics	Medication status	Methodology
Greenberg et al. (2015)	↓ RPE signal in right striatum in MDD; striatal PE-related signal associated with anhedonia severity	148 MDD, 31 HC	Unmedicated	fMRI
Keren et al. (2018)	↓ striatal activation during reward anticipation and blunted FRN response in MDD; longitudinal studies suggest these effects precede onset of depression in adolescents	Meta-analysis of 38 fMRI studies and 12 EEG studies	Mixed status	EEG and fMRI
Kumar et al. (2018)	↓ RPE signal in striatum in MDD; ↓ VTA-striatal connectivity during feedback; both striatal RPE signal blunting and habenula PPE signal associated with number of MDEs	25 MDD, 26 HC	Unmedicated	fMRI
Zhang et al. (2013)	↓ striatal activation during reward anticipation and feedback in MDD, ↑ activation in middle frontal gyrus and dorsal anterior cingulate during reward anticipation in MDD	Meta-analysis of studies including 341 MDD, 367 HC	Mixed status	fMRI
Rothkirch et al. (2017)	No differences in RPE signals in striatum and anterior insula; ↓ RPE signaling in orbitofrontal cortex in MDD; RPE signals in striatum and orbitofrontal cortex negatively associated with anhedonia severity	28 MDD, 30 HC	Unmedicated	fMRI
Rutledge et al. (2017)	No differences in RPE signals in striatum	32 MDD, 20 HC	Medicated	fMRI

*EEG, electroencephalography; fMRI, functional magnetic resonance imaging; FRN, feedback-related negativity; HC, healthy control; MDD, major depression; MEG, magnetoencephalography; PE, prediction error; PPE, punishment prediction error; RPE, reward prediction error; VTA, ventral tegmental area.*

As with MMN response changes accompanying depression, an important clinical question is whether differences in striatal activity or other aspects of reward processing are associated with depressive symptomatology. Recent research suggests that blunting of striatal RPE signaling is associated with the number of depressive episodes reported by patients, indicating that MDD has an increasing impact on reward learning processes over time (Kumar et al., 2018). Similarly, signals in the habenula have also been correlated with the number of depressive episodes experienced by MDD patients (Kumar et al., 2018). A recent meta-analysis of studies using reward tasks in depression found that blunting of both striatal activation and the FRN response were associated with depressive symptomatology, though changes in the metrics that accompanied symptom severity did not reach levels that would be useful for clinical prediction (Nielson et al., 2021). Taken together, these findings suggest that RPE signaling deficits, as indexed by reductions in striatal activation and the FRN component of the M/EEG, are potentially useful biomarkers of MDD; nevertheless, more research is warranted to determine whether these brain circuitry changes may play a causal role in the development of depression (Nielson et al., 2021).

As previously noted in relation to the MMN response, inconsistencies in neurophysiological RPE findings in MDD may be due to a number of factors, including sample size and recruitment criteria, medication status, and differences in reward tasks and incentives and punishments. In addition, the heterogeneity of findings on RPEs in MDD may also be explained by underlying heterogeneity in MDD symptomatology. The severity of anhedonia, in particular, might be a useful construct for analyzing RPE signals in this context. One study that included anhedonia in its framework found that, for MDD patients, higher levels of anhedonia were associated with reduced RPE signals in the ventral striatum and medial orbitofrontal cortex (Rothkirch et al., 2017). Another study found that among those with and without an MDD diagnosis, severity of anhedonia moderated the relationship between reward expectancy and RPE signaling in ventral striatum; this suggests that those with worse symptoms of anhedonia may experience deficits in related aspects of reward learning regardless of diagnosis (Greenberg et al., 2015). Such subtyping work has also demonstrated that resting-state hyperconnectivity between thalamic and frontostriatal networks, including the reward-related circuitry discussed here, is associated with a depressive biotype characterized by increased anhedonia and psychomotor retardation (Drysdale et al., 2017).

As research in this area expands and our neuroscientific understanding of predictive coding deficits in depression is refined, it is important to consider the real-world applications of this work. For example, a better understanding of RPE signaling in reward tasks might increase our understanding of the neural processes that mediate anhedonia and negative bias, allowing the development of more refined and better targeted pharmaceutical and psychotherapeutic interventions. Given the connection between altered reward learning and decision making processes, this research also has implications for suicide-related interventions (Dombrovski et al., 2013).

## Antidepressant Drugs and Predictive Processes

Presently, most approved antidepressant drugs target the monoaminergic system and regulate the reuptake, metabolism, or receptor pharmacodynamics of the neurotransmitters 5-HT and norepinephrine (also called noradrenaline). The typical onset of beneficial drug effects for these antidepressants takes several weeks (Quitkin et al., 1984; Gelenberg and Chesen, 2000), though mounting evidence suggests that earlier clinical and cognitive processing changes may help predict treatment outcomes (Katz et al., 1996; Harmer et al., 2009). More recently, the glutamatergic modulator ketamine has gained attention as a novel therapeutic that produces rapid-acting antidepressant effects in individuals with treatment-resistant MDD that manifest within hours of administration and last days (Zarate et al., 2006; Kishimoto et al., 2016). Concomitantly, in 2019 the FDA approved esketamine (the intranasally-administered *S*-enantiomer of ketamine) as an adjunctive treatment option for depression. This section explores the current state of the literature regarding the role of monoaminergic and glutamatergic [particularly via the N-methyl-D-aspartate (NMDA) receptor] signaling in predictive coding processes. Where appropriate, research evidence that highlights the effects of monoaminergic and glutamatergic antidepressant therapeutics on these processes in both MDD patients and healthy participants is also presented.

### Monoaminergic Drugs

5-HT, norepinephrine, and dopamine are monoamines involved in a wide range of physiological and homeostatic processes. 5-HT, for example, has been implicated in a range of behaviors, including regulating the sleep-wake cycle and hormonal levels as well as influencing cognition, sensorimotor behaviors, and emotions (Jacobs and Azmitia, 1992). Norepinephrine has been implicated in regulating arousal and adapting network activity by influencing neuromodulatory neurons and peripheral arousal levels to support adaptive, flexible behavioral responses (Sara and Bouret, 2012).

Pre-attentive sensory processing research suggests that 5-HT is important for salience detection and potentially regulates the speed of change detection during sensory tasks (Kähkönen et al., 2005). In the primary visual cortex, for instance, the distribution of 5-HT-ergic axons appears to be highest in input layer IV of the cortex (Kosofsky et al., 1984; Morrison and Foote, 1986). In contrast, while 5-HT-ergic axons are consistently found in layer IV in primary auditory and somatosensory cortices, the distribution does not appear to be preferential (Wilson and Molliver, 1991a,b). Despite variability in the distribution of 5-HT-ergic axons across sensory modalities, studies have consistently shown that 5-HT modulates the salience of sensory inputs across modalities (Jacob and Nienborg, 2018). While the role of 5-HT in salience detection has been well documented, its role in the MMN response is less clear. In healthy participants, studies using acute tryptophan depletion (ATD)—which rapidly reduces the amino acid precursor of 5-HT and 5-HT metabolite concentrations in cerebrospinal fluid—have produced mixed findings. While some studies reported that ATD increased MMN amplitudes and reduced latencies (Kähkönen et al., 2005), other studies found either reduced MMN amplitudes (Ahveninen et al., 2002) or no differences in MMN responses following ATD (Leung et al., 2009). These discrepancies might be due to methodological differences in preprocessing approaches and other analytical techniques, including choices related to M/EEG source localization techniques (Fusar-Poli et al., 2006). In addition, while ATD is thought to reduce 5-HT release and subsequently blunt neurotransmission, there is no direct evidence that it decreases extracellular 5-HT concentrations. Caution is thus needed when interpreting its selective 5-HT effects (van Donkelaar et al., 2011).

The role of 5-HT in reward processing is less clear than for sensory deviance detection, and our current understanding derives from the observation that 5-HT has an opponent relationship with dopamine (Kapur and Remington, 1996; Daw et al., 2002). Phasic levels of dopaminergic activity are known to signal positive and negative RPEs related to how different the current reward is from ongoing predictions of long-running rewards (Schultz et al., 1997; Schultz, 2016b). Given the opponent relationship between dopamine and 5-HT, one theory regarding 5-HT’s role in reward signaling is that phasic levels of 5-HT signal punishment prediction errors related to how different the current punishment is from ongoing predictions of future punishment (Daw et al., 2002). An extension of this model also accounts for how tonic 5-HT levels may represent the opportunity costs of waiting to avoid punishments (Cools et al., 2011). Studies using ATD and reward learning tasks in healthy participants have produced mixed findings, echoing studies that used MMN response tasks. One review of 36 studies that used ATD during reward learning tasks reported that lower 5-HT levels resulted in reduced sensitivity to punishments in nine of the 36 studies, with the authors noting that further research was warranted to clarify the role of 5-HT in reward tasks (Faulkner and Deakin, 2014). Similar caution should be used when interpreting results regarding 5-HT’s role in reward processing, as previously discussed in relation to sensory deviance detection.

Research examining the role of norepinephrine in sensory processing indicates that it plays a complex modulatory role in sensory signaling (Jacob and Nienborg, 2018). Norepinephrine innervation in the somatosensory cortex is both uniform and dense across cortical layers (Morrison et al., 1982; Lewis et al., 1987). Unlike 5-HT, however, norepinephrine innervation in primary auditory and visual cortices is sparse across layers and virtually absent in layer IV (Foote and Pineda, 1993). Given the sparse distribution of norepinephrine receptors in auditory and visual cortices, norepinephrine’s primary role in sensory processing appears to be in modulating NMDA receptor-mediated glutamate responses (Devilbiss and Waterhouse, 2000), gating long-term plasticity (LTP) of glutamatergic synapses, and increasing the gain of local inhibitory synapses (Salgado et al., 2016). Because norepinephrine plays only an indirect role in sensory cortex signaling via modulation of glutamatergic mechanisms, little research has examined its specific role in sensory deviance detection.

While norepinephrine’s role in sensory processing is understudied, recent work has begun to examine its role in reward-related tasks. In particular, recent evidence suggests that norepinephrine plays a role in modulating glutamatergic synapses in the nucleus accumbens, and that it might tune feedforward inhibition and impact reward-related circuitry as well as motivational states (Manz et al., 2021). Animal studies have also suggested that norepinephrine is associated with the amount of effort required to perform a reward task (here, force exerted on a grip in order to receive a reward) and that this effort is distinct from reward sensitivity (Varazzani et al., 2015; Borderies et al., 2020). While norepinephrine’s role in reward-related tasks is also understudied, current findings suggest that it plays an important role in modulating motivation and effort levels. One particularly relevant area for future research regarding where motivation and effort influence reward-related behavior would be determining the opportunity costs associated with seeking or avoiding rewards and punishments. Perhaps norepinephrine and 5-HT operate synergistically in this regard to support motivated behaviors to continue to seek rewards or avoid punishments.

Given the proposed role of 5-HT and norepinephrine in predictive coding processes, it is useful to consider the effect of antidepressant drugs that target the monoaminergic system on sensory deviance detection and reward processing. While many previously reviewed studies of the MMN response and reward processing included medicated patients, the heterogeneity of medications and the inclusion of both medicated and unmedicated samples makes it difficult to tease apart the role that specific neurotransmitters may have played in predictive processing. Another way to approach this experimentally is to give antidepressant drugs to healthy participants. Such studies found that drugs such as escitalopram, the therapeutically-active *S*-enantiomer of citalopram [a highly selective serotonin reuptake inhibitor (SSRI)], increase MMN amplitudes in healthy participants (Oranje et al., 2008; Wienberg et al., 2010). In addition, research with citalopram using appetizing and aversive food picture stimuli found reduced ventral striatum and ventral medial/orbitofrontal cortex activation in healthy participants to appetizing foods such as chocolate (McCabe et al., 2010). Research with reboxetine, a norepinephrine reuptake inhibitor, found increased neural responses in the medial orbitofrontal cortex to the same appetizing foods (McCabe et al., 2010). One difficulty with using such dosing studies to inform our understanding of how predictive coding might be altered in MDD is that these antidepressants have a delayed onset of action of several weeks. Research on antidepressant drugs that selectively downregulate the 5-HT or norepinephrine transporter found that they produced a marked loss of binding sites for the targeted neurotransmitter over an overlapping 2–3 week time window corresponding with antidepressant efficacy (Frazer and Benmansour, 2002). Some drug studies have tried to account for this delay by having healthy participants take such drugs over several days (e.g., 7 days) (McCabe et al., 2010), while other studies have examined drug effects after only a single dose (Oranje et al., 2008; Wienberg et al., 2010). One study of the serotonin-norepinephrine reuptake inhibitor (SNRI) duloxetine in healthy participants used a 2-week daily dosing regimen and found increased ventral striatum responses during a reward task (Ossewaarde et al., 2011). Further work is needed using this longer-term dosing approach that overlaps with antidepressant response to the drug in order to better characterize the roles of 5-HT and norepinephrine in predictive coding processes. As a final point, recent research has begun to explore the antidepressant efficacy of “classic” 5-HT-ergic psychedelics including psilocybin and lysergic acid diethylamide-25 in MDD patients. Examining how such drugs impact both the MMN response and reward processing are promising directions for future research.

Finally, a new class of antidepressant drugs target dopamine, in addition to 5-HT and norepinephrine. Therefore, it is useful to consider the role of dopamine in predictive processes related to the MMN response and reward-related signaling. The role of dopamine in sensory signaling is understudied, though it is thought to play an important role in modulating human attention and arousal (Coull, 1998). Limited research examining the effects of haloperidol, a partially selective dopamine D2 receptor antagonist, demonstrated that drug administration did not affect the source location or amplitude of the MMN response in healthy participants, suggesting that dopamine does not have a role in sensory deviance detection processes *per se* (Kähkönen et al., 2002). However, the drug was found to influence the amplitude of the MMN response in healthy participants during a condition where participants selectively attended to one of two simultaneously presented auditory streams, suggesting that dopamine plays a specific role in the involuntary detection of task-irrelevant deviants (Kähkönen et al., 2001). Studies examining the MMN response in healthy participants following acute tyrosine/phenylalanine depletion also suggest that reducing dopamine neurotransmission has no effect on the MMN response (Leung et al., 2009). Taken together, these findings suggest that dopamine is not directly involved in predictive coding processes at the level of sensory inputs.

Much more is known about dopamine’s role in reward processing and RPE signaling, as has been previously discussed (Matsumoto and Hikosaka, 2007; Schultz, 2007; Matsumoto and Hikosaka, 2009b; Schultz, 2016b). However, while animal models consistently demonstrate that dopamine signals code RPEs (Schultz, 2016a), research examining pharmacological manipulations of dopamine in healthy participants offer mixed findings. While dopamine antagonism has been demonstrated to consistently decrease reward learning, dopamine antagonism and dietary manipulations of dopamine offer mixed results (Webber et al., 2021). These discrepancies could be due to a number of factors including drug manipulations, dosing regimens, or the possibility that there is an optimal level of dopamine for reward-related learning, with increases beyond this level impairing reward-related functioning (Vaillancourt et al., 2013). Further research is needed in this area to elucidate how changes in dopamine signaling concomitant with depression are associated with RPE signals.

### Glutamatergic Drugs

Glutamate is the primary excitatory neurotransmitter in the brain and is important for regulating cortical excitability and experience-dependent synaptic plasticity and LTP. Glutamatergic signaling deficits have been widely reported in mood disorders including MDD (Choudary et al., 2005; Yüksel and Öngür, 2010; Bernard et al., 2011), and subanesthetic doses of the non-competitive NMDA receptor antagonist ketamine have been shown to rapidly reduce depressive symptoms (Zarate et al., 2006; Kishimoto et al., 2016). Antidepressant response to ketamine appears to rely on both high affinity antagonistic binding properties at the NMDA receptor and alpha-amino-3-hydroxy-5-methyl-4-isoxazolepropionic acid (AMPA) throughput modulation (Maeng et al., 2008; Zanos et al., 2016). In the context of predictive coding signaling, this is particularly relevant because AMPA and NMDA receptors may support distinct contributions to feedforward and feedback signaling. For example, in the visual system, AMPA receptors are primarily thought to propagate visual activity from lower to higher-order visual areas, while NMDA receptors modulate recurrent connections (Lumer et al., 1997; Dehaene et al., 2003; Self et al., 2012). Much of what is known about the NMDA receptor’s role in the MMN response and reward processing comes from pharmacological ketamine studies.

Subanesthetic-dose ketamine has been used to model schizophrenia-like effects in healthy participants, and findings from these studies can inform our understanding of the NMDA receptor’s role in sensory deviance detection. In healthy participants, ketamine administration consistently diminished auditory ERP amplitudes during drug infusion (Rosburg and Kreitschmann-Andermahr, 2016; Harms et al., 2021). Electrophysiological findings have also demonstrated that ketamine increases the latency of MMN responses in healthy participants (Umbricht et al., 2000; Kreitschmann-Andermahr et al., 2001), though its effect on amplitude is stronger than its effect on latency (Rosburg and Kreitschmann-Andermahr, 2016). In the context of the MMN response, ketamine administration was found to reduce frontal MMN amplitudes immediately post-infusion and again at 2 h post-infusion in MDD patients; furthermore, immediate change in MMN amplitude predicted antidepressant response (de la Salle, 2022). In contrast, other studies that used a roving auditory oddball task collected 3–4 h post-ketamine infusion in MDD patients found that ketamine administration increased MMN response, but only when all repetitions of the post-deviant tone were analyzed (Sumner et al., 2020). The same study found that feedforward connectivity from the primary auditory cortex to the inferior temporal cortex for the deviant tones was associated with antidepressant response. Taken together, the evidence suggests that ketamine administration consistently and acutely attenuates MMN amplitude and increases its latency in healthy participants, but that findings regarding how ketamine influences MMN response in MDD patients are mixed. Some of this discrepancy could be related to differences in the oddball task design or could be related to differences in the timing of MMN response measurements relative to ketamine administration. Additional work is needed to examine both acute and delayed ketamine effects on MMN response, particularly in unmedicated patients, in order to tease apart transient effects that result from NMDA receptor blockade from more delayed antidepressant effects that result from changes in synaptic efficacy.

Ketamine has also been administered to healthy participants during reward learning tasks. Results indicated that acute subanesthetic ketamine administration attenuated ventral striatum activation during reward anticipation (Francois et al., 2016). Additional research focused on ketamine’s effects on reward processing in MDD patients, in part spurred by recent findings that ketamine blockade of NMDA receptor-dependent bursting activity in the lateral habenula mediated antidepressant response in animal models, with subsequent disinhibitory effects in downstream reward centers (Yang et al., 2018a; Cui et al., 2019). A recent study of unmedicated MDD patients currently in remission found that ketamine increased activation in the nucleus accumbens, putamen, insula, and caudate 2 h post-administration, during the reward feedback period (Kotoula et al., 2021). Another study of medicated MDD patients found that ketamine administration resulted in increased ventral striatum and orbitofrontal cortex activation during both the reward anticipation and feedback periods of a reward task administered 1 day post-infusion (Sterpenich et al., 2019). Taken together, these findings suggest that ketamine improves sensitivity to rewards as indexed by increased activation in the striatum and other reward-related circuitry, and that these effects might be mediated by changes in NMDA receptor-mediated bursting within the lateral habenula. Additional work is needed to examine how changes in activity within these reward-related regions post-ketamine may be associated with antidepressant response in MDD.

## Conclusion

The predictive coding framework conceptualizes perception as a constructive process where internal generative models are used to predict incoming sensory inputs and their causes. MDD has traditionally been viewed as a disorder characterized by negative cognitive biases, and these biases could result in disrupted prediction error signaling within this framework. This paper reviewed the evidence for disrupted predictions in MDD in relation to both sensory deviance detection and reward processing and examined the role of 5-HT, norepinephrine, and NMDA receptor-mediated glutamate signaling in these predictive processes. While the evidence suggests that MDD is accompanied by changes in both sensory deviance detection and reward processing, much additional work is needed. Future studies should pay particular attention to medication status in MDD in order to control for the influence of antidepressant drugs on effects of interest. More work is also needed to understand how cardinal symptoms of MDD such as anhedonia and negative bias are associated with reward-related neural processing in particular. Finally, additional studies are needed to understand how 5-HT, norepinephrine, and NMDA receptor-mediated glutamate signaling might synergistically support predictive signaling in both healthy participants and MDD patients.

## Author Contributions

All authors listed have made a substantial, direct, and intellectual contribution to the work, and approved it for publication.

## Conflict of Interest

CZ was listed as a co-inventor on a patent for the use of ketamine in major depression and suicidal ideation; as a co-inventor on a patent for the use of (2R,6R)-hydroxynorketamine, (S)-dehydronorketamine, and other stereoisomeric dehydroxylated and hydroxylated metabolites of (R,S)-ketamine metabolites in the treatment of depression and neuropathic pain; and as a co-inventor on a patent application for the use of (2R,6R)-hydroxynorketamine and (2S,6S)-hydroxynorketamine in the treatment of depression, anxiety, anhedonia, suicidal ideation, and post-traumatic stress disorders. He has assigned his patent rights to the U.S. Government but will share a percentage of any royalties that may be received by the government. The remaining author declares that the research was conducted in the absence of any commercial or financial relationships that could be construed as a potential conflict of interest.

## Publisher’s Note

All claims expressed in this article are solely those of the authors and do not necessarily represent those of their affiliated organizations, or those of the publisher, the editors and the reviewers. Any product that may be evaluated in this article, or claim that may be made by its manufacturer, is not guaranteed or endorsed by the publisher.
